# Magnetic–plasmonic Ni@Au core–shell nanoparticle arrays and their SERS properties†

**DOI:** 10.1039/c9ra10354f

**Published:** 2020-01-14

**Authors:** Lu Wang, Zuobin Wang, Li Li, Jingran Zhang, Jinyun Liu, Jing Hu, Xiaomin Wu, Zhankun Weng, Xueying Chu, Jinhua Li, Zhongliang Qiao

**Affiliations:** International Research Centre for Nano Handling and Manufacturing of China, Changchun University of Science and Technology Changchun 130022 China wangz@cust.edu.cn lil@cust.edu.cn +86 431 85582925 +86 431 85582926; Key Laboratory of Laser Technology and Optoelectronic Functional Materials of Hainan Province, School of Physics and Electronic Engineering, Hainan Normal University Haikou 571158 China qzhl2007@hotmail.com +86 898-65861468 +86 898 65861468; Ministry of Education Key Laboratory for Cross-Scale Micro and Nano Manufacturing, Changchun University of Science and Technology Changchun 130022 China; College of Information Engineering, North China University of Science and Technology Tangshan 063210 China; School of Science, Changchun University of Science and Technology Changchun 130022 China

## Abstract

In this paper, large-area magnetic–plasmonic Ni@Au core–shell nanoparticle arrays (NPAs) with tunable compositions were successfully fabricated by a direct laser interference ablation (DLIA) incorporated with thermal dewetting method. The magnetic properties of the Ni@Au core–shell NPAs were analyzed and the saturation magnetization (*M*_s_) of the Ni_80_@Au_20_ nanoparticles was found to be higher than that of nickel-only nanoparticles with the same diameter. Using Rhodamine 6G (R6G) as a Raman reporter molecule, the surface enhanced Raman scattering (SERS) property of the Ni@Au core–shell NPAs with a grain size distribution of 48 ± 42 nm and a short-distance order of about 200 nm was examined. A SERS enhancement factor of 2.5 × 10^6^ was realized on the Ni_50_@Au_50_ NPA substrate, which was 9 times higher than that for Au nanoparticles with the same size distribution. This was due to the enhanced local surface plasmon resonance (LSPR) between the ferromagnetic Ni cores and the surface polariton of the Au shells of each nanoparticle. The fabrication of the Ni@Au core–shell NPAs with different compositions offers a new avenue to tailor the optical and magnetic properties of the nanostructured films for chemical and diagnostic applications.

## Abstract 2

In this paper, large-area magnetic–plasmonic Ni@Au core–shell nanoparticle arrays (NPAs) with tunable compositions were successfully fabricated by a direct laser interference ablation (DLIA) incorporated with thermal dewetting method.

## Graphical abstract

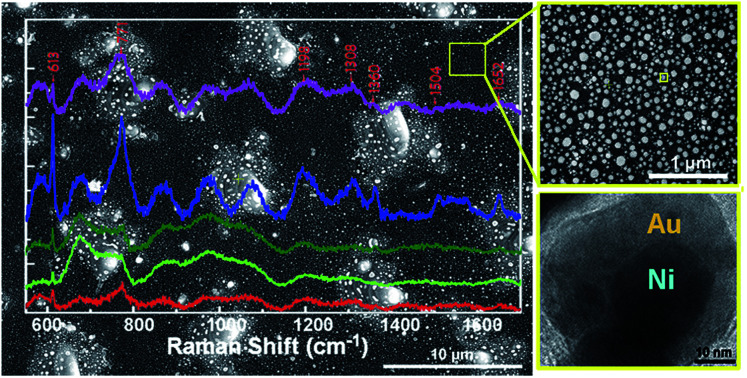

## 1. Introduction

Gold nanoparticles exhibit fascinating bio-compatibility and optical properties owing to their strong localized surface plasmon resonance (LSPR), and are incredibly useful in a wide range of chemical and biological applications, such as biomedicine,^1–3^ biological optical imaging,^4^ sensing,^5^ energy,^6^ catalysis^7^ and surface enhanced Raman scattering (SERS).^8^ Among these applications, large-area Au nanoparticle arrays (NPAs) are good SERS substrates as an attractive tool for measuring a small number of molecules with high sensitivity.^9–13^ The resonant interactions of the Au nanoparticles with incident electromagnetic radiation facilitate enhanced Raman scattering. With the development of plasmonic metamaterials and magnetic metamaterials in recent years,^14–16^ magnetic–plasmonic core–shell nanoparticles composed of magnetic cores with plasmonic shells, are currently the subject of research to achieve multifunctionality in a single material.^17–19^ They have shown a number of outstanding plasmonic resonance effects as well as magnetic properties.^20^ Meanwhile, coating pure magnetic metals with Au shells provides a surface layer with relatively inert chemical properties that can maintain the magnetic properties of the core materials and increase biological compatibility.

Different synthetic methods have been developed for the fabrication of bimetallic particles.^21,22^ The common techniques include wet chemical synthesis, photochemical synthesis, sputter deposition, electroless plating, and electrochemical synthesis. Different nanostructures can be obtained depending on the applied conditions. However, the final nanostructures synthesized by chemical methods are normally colloidal and the synthesis usually contains multi steps. Besides, additional steps of assembly may be required for the NPAs to make the particle monolayer on a substrate. Recently, different methods have been reported to fabricate magnetic–plasmonic nanostructure arrays for specific purpose. Ni–Au bimetallic NPAs were fabricated by laser annealing of bimetallic thin films.^23^ Fe_3_O_4_@Au-NP magnetic field induced assembly was used for the fabrication of Fe_3_O_4_@Au@Ag nanoflower chains.^24^ Ultrathin alumina mask technique was used during the deposition of Ni and Au for the synthesis of Ni/Au hybrid NPAs as a SERS substrate.^25^ Despite these efforts, an appropriate method for fabricating high-throughput large-area magnetic–plasmonic core–shell NPA substrates with high SERS enhancement and excellent reproducibility remains a fundamental challenge.

We have previously introduced an innovative approach to generate ordered Ni–Au NPAs on the laser interference-structured substrates by thermal dewetting.^26^ The direct laser interference ablation (DLIA) enables the formation of periodic patterns with defined long-range ordered bimetallic nanoparticles on the templates. The research has shown that bimetallic NPAs fabricated by DLIA-templated dewetting method are reliable and controllable. Therefore, a series of Ni@Au core–shell NPAs with different compositions of Au and Ni were synthesized in this work. The effect of synthesis conditions on the formation of core–shell nanoparticles were investigated and the core–shell structure of nanoparticles was characterized in detail. Furthermore, the magnetic properties of the Ni@Au core–shell NPAs were analyzed and compared with the nickel-only NPAs. Finally, SERS measurement results of R6G on the as-prepared Ni@Au core–shell NPA substrates were compared to observe the dependence of SERS intensity on the metal compositions.

## 2. Experimental section

### 2.1 Direct laser interference ablation

**Fig. 1:**
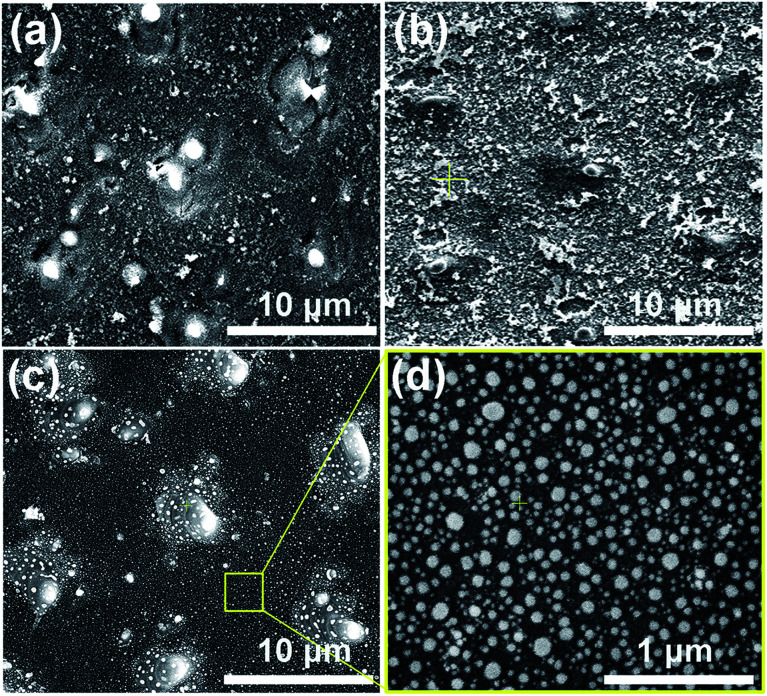
(a) SEM images of the as-ablated silicon surface, (b) corresponding silicon surface with a 20 nm Ni–Au bilayer film deposition, (c and d) corresponding Ni_50_Au_50_ NPAs formed after the vacuum rapid thermal annealing of 950 °C for 5 min.

In this experiment, a nanosecond laser (Innolas) three-beam interference ablation system with a wavelength of 1064 nm, a frequency of 10 Hz and a pulse duration of 7 ns was employed for the DLIA (for more detail see our previous work in ref. 26). The geometric configuration of three beams is an equilateral triangle and the incident angle is 4.1°. The laser interference fluence of 300 mJ cm^−2^ and the pulse number of 30 were used to process all silicon samples. All the samples used in the experiment were monocrystalline p-doped silicon wafers. The morphology of the as-ablated silicon substrates was shown in Fig. 1(a).

### 2.2 Bilayer deposition

The Ni–Au bilayer of 20 nm (10 nm Ni and 10 nm Au) was deposited onto the as-ablated silicon substrates utilizing a Turbo sputter coater in which the Au was chosen as the top layer. And the surface morphology of the silicon substrate after the film deposition was shown in Fig. 1(b).

### 2.3 Vacuum rapid thermal annealing

Vacuum rapid thermal annealing (temperature 950 °C, time 1 to 10 min) was used to bring on the dewetting of Ni–Au bilayer films on all the as-deposited substrates. The as-annealed Ni_50_Au_50_ NPAs were finally prepared after the heating time of 5 min and subsequent fast cooling process, as depicted in Fig. 1(c and d). A large-area Ni_50_Au_50_ NPA SEM image was also presented (ESI Fig. S1†).

Based on our previous study, the Ni–Au NPAs with different compositions were able to be synthesized by changing the initial composition of the bilayer. The volume ratio of Au to Ni was adjusted while the thickness of the bilayer was 20 nm, so that a series of NPAs were successfully prepared as Ni_100_, Ni_80_Au_20_, Ni_60_Au_40_, Ni_50_Au_50_, Ni_40_Au_60_, Ni_20_Au_80_ and Au_100_, respectively. As per previous study, it was found that the size and density of the annealed particle as well as the pattern of the arrays were able to be controlled by the applied laser-interference parameters.^26^ Thus, we processed all the substrates with the same DLIA parameters, including the laser interference fluence, pulse number and their geometric configuration, so that the Ni–Au NPAs of different compositions obtained after annealing have the same morphology as Ni_50_Au_50_, as depicted in Fig. 1(c and d).

### 2.4 SERS measurements

SERS measurements were performed using Rhodamine 6G (R6G) as molecular probes. A micro-Raman spectroscopic system (labRAM HR E, HORIBA) equipped with a 532 nm laser (CNILASER MLL-532) and a 50× focusing objective was used to characterize the SERS properties. The laser power of approximate 1 mW and beam diameter of 1 mm were used to excite the samples. Seven types of samples with Ni NPAs, Ni–Au NPAs and Au NPAs on the substrates were immersed in the 10^−4^ M and 10^−6^ M R6G aqueous solution for 30 min, respectively. A gentle nitrogen flow was used to dry the samples after that. Three locations on each sample were averaged for each Raman and SERS spectrum presented.

### 2.5 Instrumentation

The morphologies of the as-prepared silicon and Ni–Au NPA substrates were imaged by a high-resolution scanning electron microscope (SEM, FEI QUANT-250 FEG). Energy-dispersive X-ray spectroscopy (EDS, Oxford Instruments) was used to analyze the element composition. *In situ* X-ray photoelectron spectroscopy (XPS) (ESCALAB 250) was applied for the investigation of the crystal structure of the final composite nanoparticles. Transmission electron microscopy (TEM) and high resolution transmission electron microscopy (HRTEM) sample observations were performed on a JEM-2100F transmission electron microscope. High-angle annular dark field (HAADF) scanning TEM images were carried out on the JEM-2100 TEM with an HAADF detector. A superconducting quantum interference device (SQUID) magnetometer (Quantum Design-MPMS-XL7) was used to perform the magnetic measurements.

## 3. Results and discussion

### 3.1 Synthesis of Ni@Au core–shell nanoparticles

**Fig. 2:**
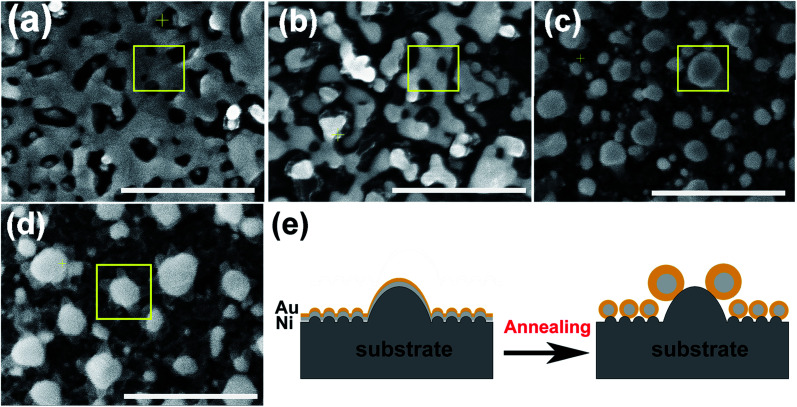
SEM images of the metal bilayer taken at different annealing times of 1 min (a), 2 min (b), 5 min (c) and 10 min (d), showing the dewetting phenomenon (scale bar = 500 nm). (e) Schematic illustration of the formation of Ni@Au core–shell nanoparticles in (a–c). The conversion of the thin bilayer into a rivulet upon dewetting, followed by an array of core–shell nanoparticles.

According to the phase diagram of Ni–Au system, at the high temperature of 800–1000 °C, a homogeneous solid fcc solution with a miscible gap across the entire concentration range was shown.^27^ Therefore, the heating times from 1 to 10 min at the temperature of 950 °C were chosen for the experiments to find the optimal synthesis conditions for the core–shell structure. Fig. 2 shows the morphology of the metal bilayer as a function of heating time. The metallic film became thinner at the edges between the nanostructures, leading to the holes in the film when the annealing begins at the heating time of 1 min, as displayed in Fig. 2(a). The retraction of the rims of the hole formed the rivulets due to the Rayleigh instability. Then, the rivulets were about to rupture and fragment to form nanoparticles at the heating time of 2 min, as shown in Fig. 2(b). We assume it occurs in the same location for the individual Ni and Au layers before the complete intermixture of the metal bilayer. As the heating times up to 5 min, the Ni and Au layers are completely intermixed, and spherical Ni–Au nanoparticles are formed, as depicted in Fig. 2(c). After that, continuous heating causes the two metals to begin to diffuse into each other and nucleate when the temperature is rapidly decreased. It is known that the diffusion of Ni crystals into Au atoms is more likely than the diffusion of Au atoms into Ni crystals.^28^ Also, the heteronucleation caused by a temperature drop will suppress the homonucleation and lead to the formation of a gold shell on the nickel core,^29^ as indicated in Fig. 2(e). However, when the time up to 10 min, the flower-like nanoparticles are formed, as displayed in Fig. 2(d). The petal-like substance around the crystalized nanoparticles is considered to be silicon oxide, which is presumably formed by the catalysis of Au at the Au/Si interface.^30,31^ The EDS mapping scans of a single crystalized Ni_50_Au_50_ nanoparticle in Fig. 2(d) were also provided (ESI Fig. S2†). It can be seen that the O–K signal at the petal locations is much higher than that at the surrounding substrate.

**Fig. 3:**
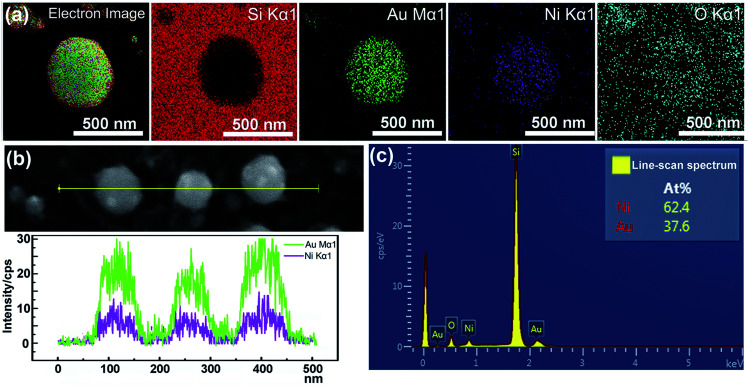
(a) EDS mapping images of an individual Ni_50_Au_50_ nanoparticle and the corresponding element mapping images of Si–K, Au–M, Ni–K and O–K. (b) EDS line-scan analysis of the nanoparticles below 100 nm showing the distribution of the two metals. (c) EDS point-scan spectrum of the particles in (b).

The EDS mapping measurement of an individual spherical Ni_50_Au_50_ nanoparticle was used to analyze the Au and Ni structure of the nanoparticle, as shown in Fig. 3(a). Considering the spatial resolution of the instrument, the larger particles on the top of the pattern are used for the composition analysis. The corresponding element mapping images show that the Au can be detected on the entire nanoparticle, and the signal is uniform in both the periphery and the center, indicating that gold mainly exists in the shell of the nanoparticle. The Ni–K signals are weak in the periphery and strong in the core, suggesting that nickel is mainly distributed in the core of the nanoparticle. Fig. 3(b) shows both the Au–M and Ni–K by line scanning the nanoparticles, which confirms the homogenous distribution of the Au and Ni, even if the annealed particles are less than 100 nm. The point-scan analysis shows the existence of C, O, Si, Au and Ni elements in the nanoparticle and on the substrate. By excluding other elements, the molar ratios of Au and Ni in the Ni_50_Au_50_ nanoparticles were calculated to be 37.6% and 62.4%, respectively, which are similar to the molar fractions of equal volumes of Au and Ni, as displayed in Fig. 3(c). The Au signal is much stronger than the Ni signal in the line/mapping scan images, which further proves that the Ni–Au hybrid nanoparticle is of the Ni@Au core–shell structure. This is also clearly confirmed by the XPS and TEM data of the Ni_50_@Au_50_ nanoparticle in the morphological and characterization part. Due to the immiscibility of the two metals, the structural properties of the resulting nanoparticles are not influenced by the change in the composition of bimetallic films.^23^ That is, the structures of the final particles are the same after thermal annealing of Ni–Au bilayer with different compositions.

### 3.2 Morphological and structural characterization

**Fig. 4:**
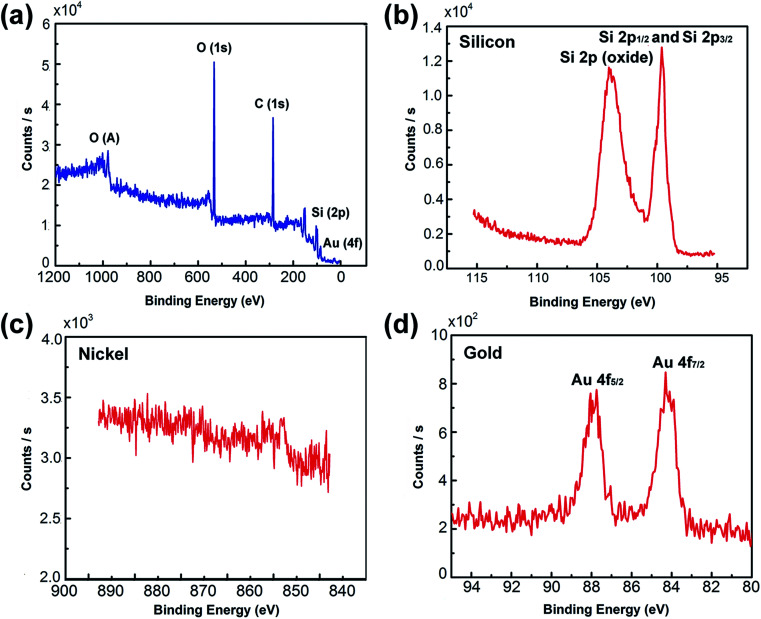
XPS spectrum of Ni_50_@Au_50_ NPA substrates. (a) XPS data acquired over a wide range of binding energies (0–1200 eV) from a Ni_50_@Au_50_ NPA substrate. (b) Detailed XPS data acquired for B.Es ranging from the silicon region of 95 to 115 eV. (c) Detailed XPS data acquired for B.Es ranging from the nickel region of 845 to 890 eV. (d) Detailed XPS data acquired for B.Es ranging from the gold region of 80 to 95 eV.

*In situ* XPS was employed to investigate the crystal phase of the Ni_50_@Au_50_ nanoparticles on the substrate, as shown in Fig. 4. The XPS spectrum verified the existing of O, C, Si and Au elements in the substrate, as displayed in Fig. 4(a). The detailed peaks of Si 2p_1/2_ and Si 2p_3/2_ at 99.4 eV, also the silicon dioxide at 103.5 eV verified the existing of silicon and silicon oxide in the substrate (Fig. 4(b)).^32^ The detailed peaks of Ni 2p_1/2_ and Ni 2p_3/2_ at 869.9 eV and 852.6 eV were hardly detected in the XPS spectra corresponding to the Ni species (Fig. 4(c)).^33^ The significant peaks of Au 4f_5/2_ and Au 4f_7/2_ at 87.7 eV and 84.0 eV, respectively were observed, which were in agreement with the standard peak positions in the stoichiometric Au (Fig. 4(d)).^34^ XPS normally probes to a depth of a few nanometers.^35^ It is assumed that the inherent surface sensitivity of XPS prevents the characteristic Ni signal from being observed for most of the particles, when the thickness of Au shell exceeds 10 nm. Only the surface composition of the Au is detected, indicating that Au atoms dominate the surface composition of the bimetallic Ni–Au nanoparticles. Therefore, the XPS spectra collected from Ni_50_@Au_50_ NPAs further demonstrate the core–shell architecture of nanoparticles.

**Fig. 5:**
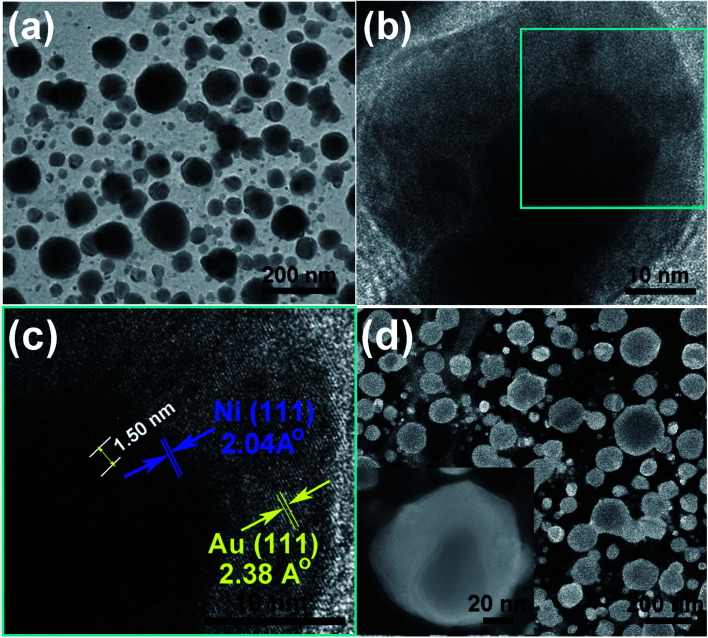
Structural characterization of the Ni_50_@Au_50_ nanoparticles. (a) The bright-field large-area TEM image of nanoparticles. (b and c) HRTEM images of an individual nanoparticle show the lattice fringes belonging to the core and shell component. (d) HAADF images of nanoparticles and single nanoparticle (inset).

In order to further confirm the core–shell structure of the as prepared Ni_50_@Au_50_ nanoparticles, TEM and HRTEM images were used for the structural and compositional characterization, as shown in Fig. 5(a–c). All the TEM samples were fabricated by peeling off the NPAs from the substrate utilizing a poly (methyl methacrylate) (PMMA) and transferring the PMMA film directly to a Cu grid.^36^ The as-annealed spherical nanoparticles shown in Fig. 5(a) have a grain size distribution of 48 ± 42 nm and a short-distance order of about 200 nm (ESI Fig. S3†). The atomic mass of gold is heavier than nickel, so as expected, the Au shells will be darker and the Ni cores will be brighter in contrast. Therefore, the difference in contrast between cores and shells is not obvious in the large-area TEM image. However, the HRTEM images of an individual nanoparticle shown in Fig. 5(b and c) give further evidence of the core–shell structure, showing that the lattice spacing of 2.04 Å is consistent with fcc-nickel (111) planes in the core and 2.38 Å is consistent with fcc-gold (111) planes in the shell.

In addition, the appearance of Moire pattern with an interval spacing of 1.50 nm was found in the core area due to the difference in the crystalline plane interval of gold shell and nickel core. HAADF imaging was carried out as a convincing method to confirm the core–shell structure. Fig. 5(d) shows the HAADF images of as-prepared Ni_50_@Au_50_ nanoparticles. An apparent contrast between the dark core and bright edge components was revealed in the single nanoparticle image (inset), which indicated the different chemical compositions in the core and shell. Since the atomic number of Au is higher than Ni and it has a stronger ability to scatter electrons, it behaves brighter in the HAADF images,^29^ which reveals a typical core–shell structure.

### 3.3 Magnetic properties

**Fig. 6:**
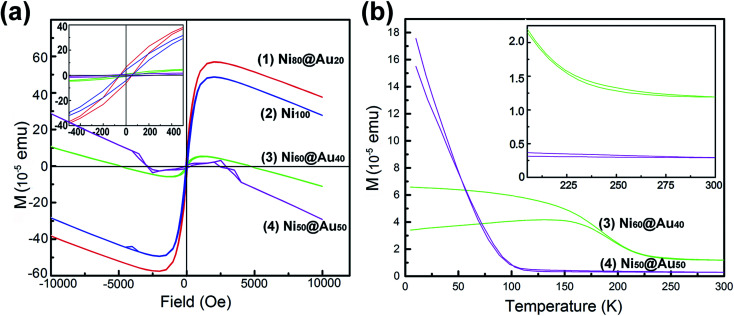
(a) Hysteresis loops of as-annealed Ni@Au core–shell NPAs with different compositions and Ni NPAs. (b) FC–ZFC magnetization *versus* temperature curves for the Ni_60_@Au_40_ and Ni_50_@Au_50_ NPAs.

The hysteresis loops (Fig. 6(a)) show the magnetization curves of Ni@Au core–shell NPAs and nickel-only NPAs on the substrate of 4 cm^2^ at 300 K. The Ni@Au core–shell NPAs are designated as Ni_80_@Au_20_, Ni_60_@Au_40_, Ni_50_@Au_50_, Ni_40_@Au_60_ and Ni_20_@Au_80_ NPAs, respectively. It is impossible to detect the magnetic property in the samples of the same area when the Ni composition less than 50%. A typical ferromagnetic behavior (inset) is shown for all measured nanoparticles. When the applied field reaches about 2500 Oe, the magnetic moment begins to saturate. The measured values of saturation magnetization (*M*_s_) for Ni_50_@Au_50_ and Ni_60_@Au_40_ NPAs are 2.0 × 10^−5^ and 6.0 × 10^−5^ emu, respectively. They are much lower than the value of nickel-only NPAs. This is because of the smaller Ni core diameter and the contribution of a high composition of diamagnetic gold shells. The *M*_s_ is improved with the increasing Ni core diameter. However, the *M*_s_ of the Ni_80_@Au_20_ NPAs was found to be higher than that of nickel-only NPAs, which was a drastic enhancement in the magnetic moments. The enhanced magnetization in Ni_80_@Au_20_ NPAs may come from the trapped metallic electrons at the Ni–Au interface.^37,38^ The chemical potential gradient generated at Ni–Au interface is able to trap the electrons from the gold atom and produce an orbital moment there. Still, a large interface is needed for the core–shell particles to produce interfacial effect.^39^ For Ni_20_@Au_80_ and Ni_40_@Au_60_ NPAs, the magnetization is undetected as a result of the small interface arising from the small Ni core in each nanoparticle.

Fig. 6(b) shows the temperature-dependent zero-field cooling (ZFC) and field cooling (FC) magnetization curves for the substrates of Ni_50_@Au_50_ and Ni_60_@Au_40_ NPAs, with an applied field of 100 Oe. The blocking temperature (*T*_B_) determined by the intersection of the ZFC and FC curves is higher than the room temperature for both Ni_50_@Au_50_ and Ni_60_@Au_40_ NPAs (inset). It evidently shows the ferromagnetism of the particles at room temperature, which is also consistent with the measured results in Fig. 6(a). The magnetocrystalline anisotropy constant (*K*_u_) was calculated by the following equation: *K*_u_ = 25*k*_B_*T*_B_/*V*,^40^ where *k*_B_ is the Boltzmann constant, and *V* is the volume of a Ni magnetic core. Since the value of *K*_u_ for fcc Ni@Au core–shell nanoparticles is constant. According to the equation, *T*_B_ of the Ni@Au core–shell nanoparticles should be proportional to the volume of Ni cores. This indicates that the value of *T*_B_ for the nanoparticles can be adjusted by changing the diameter of the Ni cores. As can be seen from the result, the *T*_B_ of the Ni_60_@Au_40_ NPAs was higher than that of Ni_50_@Au_50_ NPAs, which is due to the increasing Ni core diameter. And, the magnetization of Ni_60_@Au_40_ NPAs is less affected by temperature fluctuations than that of Ni_50_@Au_50_ NPAs, as indicated in Fig. 6(b). Both interface magnetism and diamagnetism contribute to the total magnetization of Ni@Au core–shell NPAs. As we know, the diamagnetism is independent of temperature. The interfacial moment is very large at low temperatures, which will overcome the diamagnetic contribution, and make the NPAs with positive *M*_s_. On the contrary, the interfacial magnetism is reduced and the diamagnetism is dominant at high temperatures, which make the NPAs with diamagnetic behavior. The negative moments for both Ni_60_@Au_40_ and Ni_50_@Au_50_ NPAs at 300 K confirm the conclusion, as shown in Fig. 6(a). Therefore, the magnetic properties of Ni@Au core–shell NPAs are adjustable with our fabrication method to suit for applications at different temperatures.

### 3.4 SERS properties

**Fig. 7:**
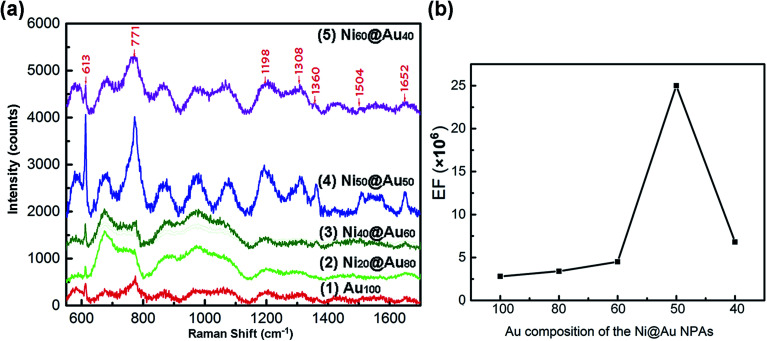
(a) SERS spectra of the 10^−6^ M R6G molecules adsorbed on the substrates of Au_100_ (1), Ni_20_@Au_80_ (2), Ni_40_@Au_60_ (3), Ni_50_@Au_50_ (4) and Ni_60_@Au_40_ (5) NPAs, respectively. (b) The dependence of the EFs on the composition of Au shell at the 613 cm^−1^ peak for the 10^−6^ M R6G molecule absorption.

Seven types of samples including gold-only NPAs, Ni@Au core–shell NPAs and nickel-only NPAs formed on the identical substrates were used for SERS measurement. The as-annealed spherical nanoparticles have a same size distribution of 48 ± 42 nm and a short-distance order of about 200 nm (ESI Fig. S3†) for all samples. Fig. 7 shows the Raman spectra of R6G molecules with a concentration of 10^−6^ M adsorbed on Au_100_ (spectrum (1)), Ni_20_@Au_80_ (spectrum (2)), Ni_40_@Au_60_ (spectrum (3)), Ni_50_@Au_50_ (spectrum (4)) and Ni_60_@Au_40_ (spectrum (5)) NPA substrates, respectively. As it can be seen, the typical peaks of R6G molecules are at 613 cm^−1^, 771 cm^−1^, 1198 cm^−1^, 1308 cm^−1^, 1360 cm^−1^, 1504 cm^−1^, and 1652 cm^−1^ corresponding to the C–C–C ring in-plane vibration mode, C–H out-of-plane bend mode, C–C stretching mode, aromatic C–C stretching mode, C–N stretching mode, aromatic C–C stretching mode and aromatic C–C stretching mode, respectively.^41,42^ Obviously, the Ni_50_@Au_50_ NPA substrate has the most significant Raman enhancement than other substrates. The spectrum of Ni_80_@Au_20_ NPA substrate shows the similar mode features and slight increase in the absorption bands, but weaker characteristic SERS peaks are generated. Finally, the nickel-only NPAs exhibit a stronger increase in the linewidth of the modes, but almost no obvious R6G vibration signals are observed (ESI Fig. S4†). The SERS spectrum measurement results of the NPAs at 10^−4^ M R6G (ESI Fig. S5†) also show similar trends to the 10^−6^ M spectrum, except for the nickel-only NPAs that are out of the measurement range due to the high fluorescence effect.

The characteristic absorption band of 613 cm^−1^ was considered for the Raman enhancement factor (EF) calculation. The EF was calculated by EF = (*I*_SERS_/*I*_NR_)(*C*_NR_/*C*_SERS_), where *I*_SERS_ and *I*_NR_ are the intensity of SERS and normal Raman spectrum at the selected Raman peak of 613 cm^−1^, respectively.^43^*C*_SERS_ and *C*_NR_ are the concentrations used for SERS substrates and Si substrate, respectively. In this work, *C*_SERS_ is 10^−6^ and *C*_NOR_ is 0.25 M with the *I*_NR_ of 225. The calculation results are shown in Table 1. And, Fig. 7(b) shows the dependence of the EFs on the composition of Au shells in particles. With the further increase of the Ni composition from 20%, 40% to 50%, the SERS signal was enhanced remarkably. However, when the composition of Ni core reaches 60%, the SERS signal no longer continues to increase but decreases instead. The largest SERS enhancement at 613 cm^−1^ occurs when the volume proportion of the Ni is 50%, and the Raman intensity of the Ni_50_@Au_50_ NPAs was ∼9 times higher compared to the gold-only NPAs. The EF was estimated to be 2.5 × 10^6^ of SERS of normal Raman measurement under the 10^−6^ M R6G solution, indicating that the Ni_50_@Au_50_ NPA substrate has an excellent SERS performance. Accordingly, it can be seen that when the size, shape and spacing of the nanoparticles are the same, the Raman enhancement effect of the Ni@Au core–shell NPAs as the SERS substrate is mainly determined by the composition of the core–shell structure.

**Table 1:** EFs of SERS intensity at 613 cm^−1^ peaks of 10^−6^ M R6G molecules absorbed on the nickel-only NPA substrate, Ni_80_@Au_20_, Ni_60_@Au_40_, Ni_50_@Au_50_, Ni_40_@Au_60_, Ni_20_@Au_80_ NPA substrates and gold-only NPA substrate

Sample	Probing molecule concentration	EF
Nickel-only	10^−6^ M	—
Ni_80_@Au_20_	10^−6^ M	—
Ni_60_@Au_40_	10^−6^ M	6.8 × 10^5^
Ni_50_@Au_50_	10^−6^ M	2.5 × 10^6^
Ni_40_@Au_60_	10^−6^ M	4.5 × 10^5^
Ni_20_@Au_80_	10^−6^ M	3.4 × 10^5^
Gold-only	10^−6^ M	2.8 × 10^5^

The SERS enhancement is mostly due to the intense electromagnetic coupling between the adjacent nanoparticles as well as the enhanced LSPR between the Ni cores and the surface polariton of Au shells in each nanoparticle.^44^ As we know, the Ni@Au core–shell NPAs exhibit ferromagnetic behavior due to an interfacial effect. It is speculated that the enhanced local electromagnetic field is generated at the Ni–Au interface, and magnified at the Au shell, which cannot be achieved in the gold-only nanoparticles. The ferromagnetism of the Ni@Au nanoparticles can affect the surface electrons and thus affect the plasma mode distribution of R6G molecules. Thus, the SERS enhancement effect of the R6G molecules is achieved, but shows a different vibrational mode than the gold-only nanoparticles, as shown in Fig. 7. For Ni_20_@Au_80_ and Ni_40_@Au_60_ NPAs, the SERS intensity is almost in the same level of gold-only NPAs due to the thick Au shell in each nanoparticle. However, as the proportion of Ni increases, the modes are significantly broadened, which is attributed to enhanced coupling from the surface modes into the adjacent absorptive Ni nanoparticles. Because the localized plasmon mode is highly sensitive to the composition of the precise material at the nanoparticle surface.^45^ Not all localized plasmons generate SERS signals with equal efficiency. For Ni_80_@Au_20_ NPAs, although the nanoparticle has a thin gold shell, the plasmonic electric field can penetrate into the adjacent Ni nanoparticles, thereby increasing absorption and reducing SERS. For nickel-only NPAs, there is only strong plasmon resonance but no Raman enhancement signal (ESI Fig. S4†). Thus, the sample of Ni_50_@Au_50_ is the optimum thickness of the Au shell for the Ni@Au core–shell nanoparticles which shows maximum SERS, beyond which, the diamagnetism of Au electrons will dominate and decrease the plasmons from the interfacial ferromagnetic behavior.

The mechanism of magnetic–plasmonic Raman enhancement can be explained by previous reports.^23,29,46–48^ As for the magnetic–plasmonic core–shell nanoparticles, the Au shell can provide a plasmon resonance optical response. The resonance frequency is determined by the geometry, dielectric properties of the nanoparticle core, and surrounding medium of the nanoparticle.^47^ In this experiment, the effect of composition of core–shell nanoparticles on SERS enhancement is realized by changing the dielectric function of the system. The presence of Ni cores causes the imaginary part of the dielectric constant of the system to increase, thereby enhancing the absorption of incident radiation.^23,48^ The characteristic surface plasmon resonance (SPR) band for Au nanoparticles is observed at 522 nm. Due to the nanocore effect, the plasmonic absorption spectrum of Ni@Au nanoparticles is red-shifted and widened. With the varying Au shell thickness, the maximum of the band can reach to about 600 nm, so that the plasmonic properties are tunable. It is reported that the SPR band of pure Ni has no maximum characteristic value.^29,46^ Thus, sufficient gold shell thickness is required to provide a more significant plasmon excitation effect, which results in more efficient Raman mode excitation.^23^ As the nickel core becomes larger, such as Ni_80_@Au_20_, the absorption increases, but the gold shell is too thin to provide sufficient plasmon excitation. Thus, Ni_50_@Au_50_ NPAs is an optimal gold shell thickness that enables it to provide a plasmon excitation effect with the highest SPR spectral absorption. However, for increasing Au shell thickness, the interaction between the core and shell decreases, resulting in no significant SERS enhancement for the Ni_20_@Au_80_ and Ni_40_@Au_60_ NPAs. As a result, the magnetic–plasmonic Ni@Au core–shell NPA demonstrates a promising SERS substrate with high stability and good magnetic capabilities, which is suitable for biological and chemical molecule detection at low concentrations.

## 4. Conclusion

In summary, the fabrication of large-area, cost-effective NPA substrates with tunable composition of Ni@Au core–shell nanostructures was presented in the paper. The magnetic properties and SERS active performance of the Ni@Au core–shell NPAs were investigated. The *M*_s_ of the prepared Ni_80_@Au_20_ nanoparticles was found to be enhanced due to the interfacial effect of the core–shell structure. The SERS result shows that the Ni_50_@Au_50_ NPA substrate has the strongest SERS enhancement of approximate 9 times of that of gold-only NPA substrate with the same size, shape and spacing. It reveals that there is an optimum size of the Au shell for the Ni@Au core–shell nanoparticle which shows the maximum plasmons generated by the interfacial ferromagnetic behavior. Better Raman enhancement achieved with less Au material confirms the cost-effectiveness of this method. In addition, the properties of NPAs can be tailored by controlling the pattern, size and composition of the core–shell nanoparticles to create nanostructured films with specific properties, providing new avenues for biosensing and chemical applications.

## Conflicts of interest

The authors declare that they have no conflict of interest.

## Supplementary Material

RA-010-C9RA10354F-s001
