# Supplementary material for: Magnetic–plasmonic Ni@Au core–shell nanoparticle arrays and their SERS properties
Source: RSC Adv. 2020 Jan 14;10(5):2661–9. doi: 10.1039/c9ra10354f (PMC9048804; doi:10.1039/c9ra10354f)
Supplement: RA-010-C9RA10354F-s001 [file RA-010-C9RA10354F-s001.pdf]

Electronic Supplementary Material (ESI) for RSC Advances.
This journal is © The Royal Society of Chemistry 2020

Supporting Information

Title:

Magnetic-plasmonic Ni@Au core-shell nanoparticle arrays and their SERS properties

Author:

Lu Wang[a,c], Zuobin Wang[a,c]*, Li Li[a,c]*, Jingran Zhang[a], Jinyun Liu[d], Jing Hu[a], Xiaomin Wu[a], Zhankun Weng[a,c], Xueying Chu[e], Jinhua Li[e] and Zhongliang Qiao[b]*

Affiliation:

[a]International Research Centre for Nano Handling and Manufacturing of China, Changchun University of Science and Technology, Changchun 130022, China

[b]Key Laboratory of Laser Technology and Optoelectronic Functional Materials of Hainan Province, School of Physics and Electronic Engineering, Hainan Normal University, Haikou 571158, China

[c]Ministry of Education Key Laboratory for Cross-Scale Micro and Nano Manufacturing, Changchun University of Science and Technology, Changchun 130022, China

[d]College of Information Engineering, North China University of Science and Technology, Tangshan 063210, China

[e]School of Science, Changchun University of Science and Technology, Changchun 130022,

China

Corresponding author:

Zuobin Wang: phone: +86 431 85582926, fax: +86 431 85582925, e-mail address: wangz@cust.edu.cn

Zhongliang Qiao: phone: +86 898 65861468, fax: +86 898-65861468, e-mail address: qzhl2007@hotmail.com

Li Li: phone: phone: +86 431 85582926, fax: +86 431 85582925, e-mail address: lil@cust.edu.cn

Figures

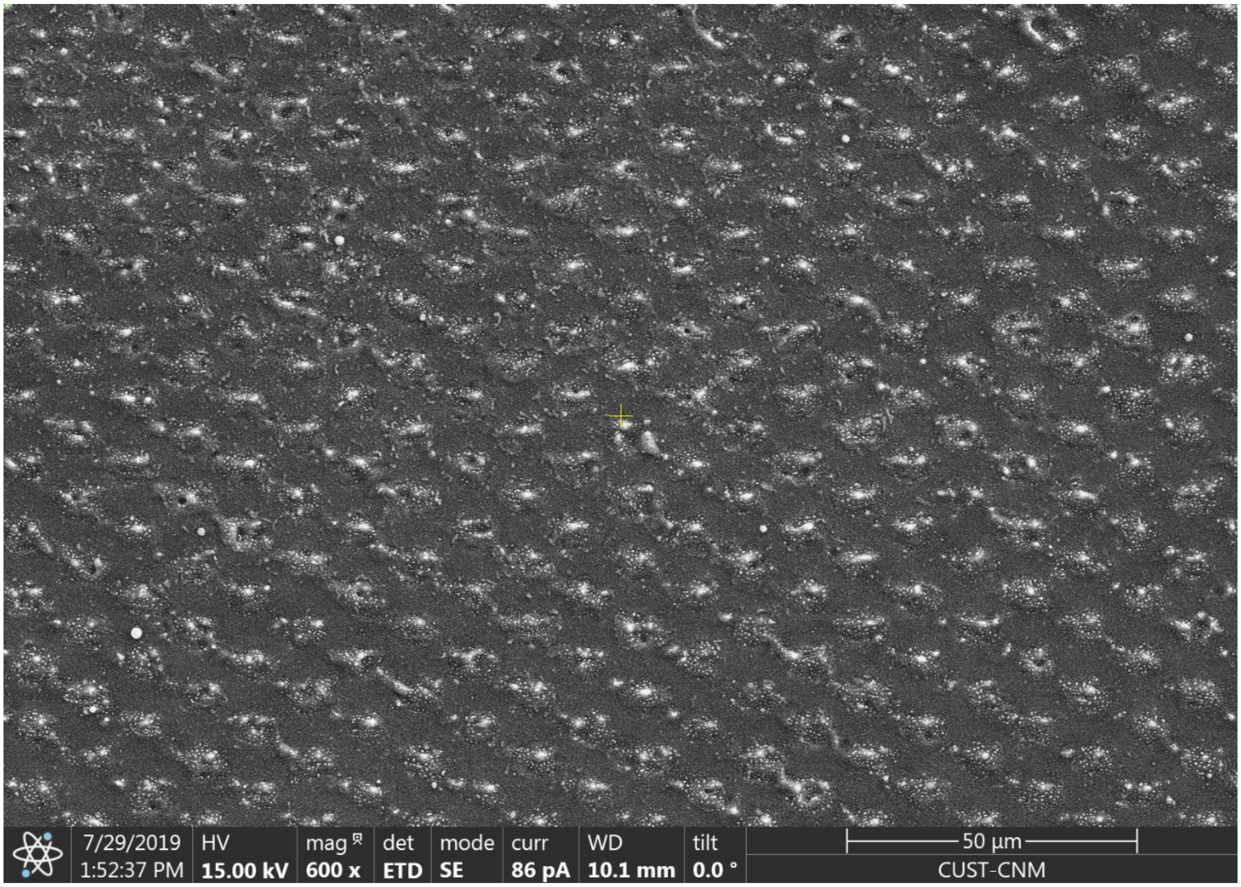

Figure S1. Low magnification SEM image of the $Ni_{50}@Au_{50}$ NPAs corresponding to Figure 1(c).

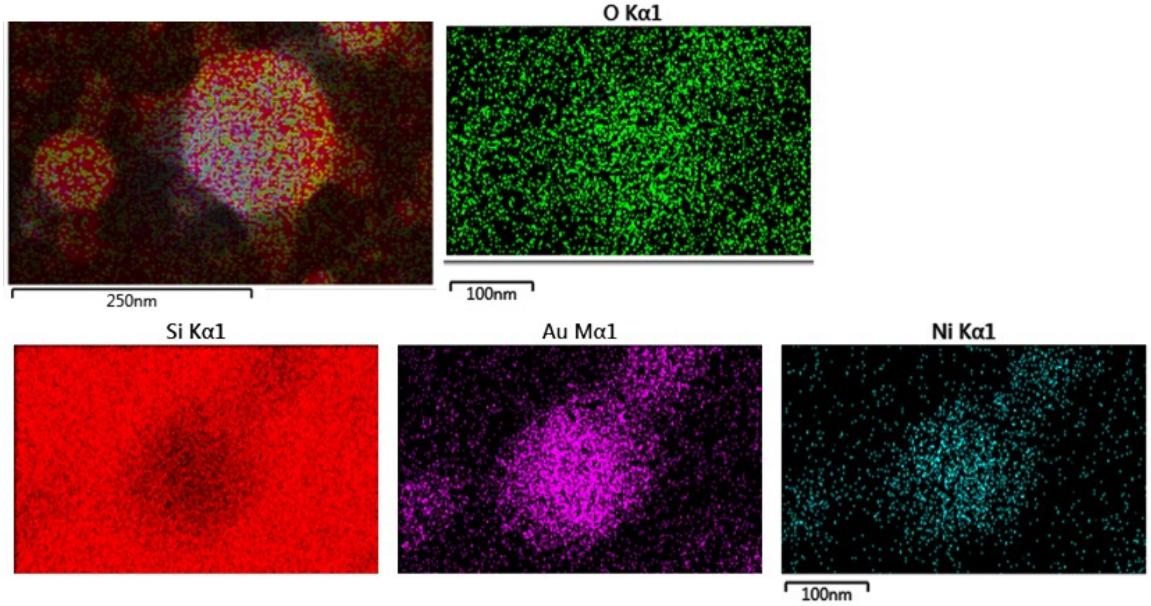

Figure S2. Higher magnification EDS mapping of single crystalized particle corresponding to Figure 2(d).

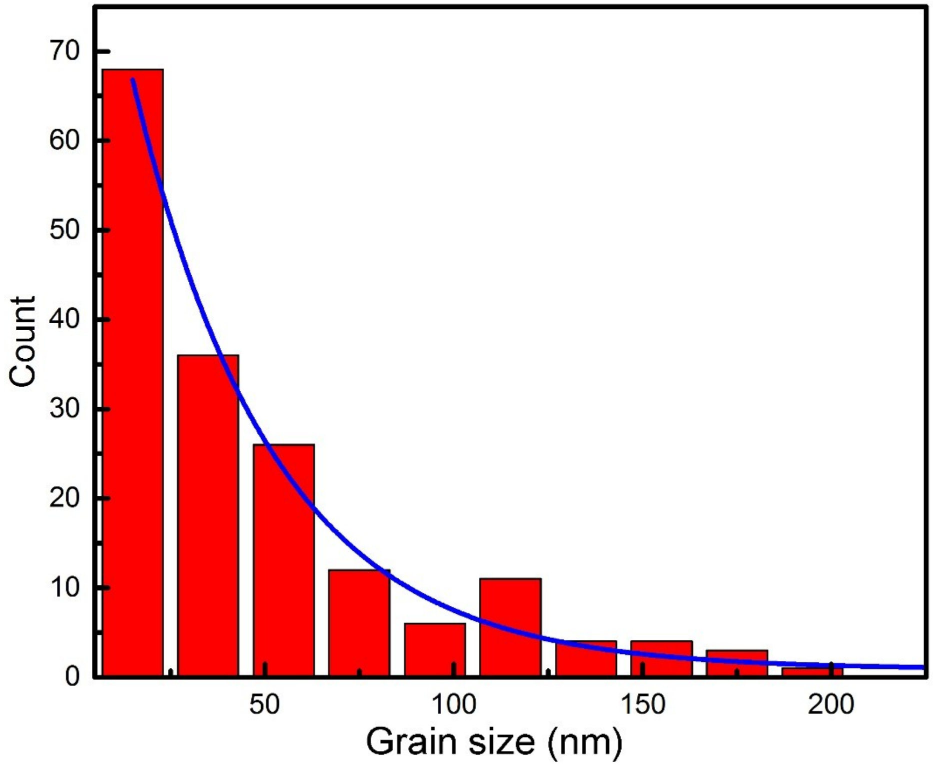

Figure S3. Plots of the grainsize distribution of the as-annealed $Ni_{50}@Au_{50}$ core–shell particles in the

TEM image of Figure 7(a).

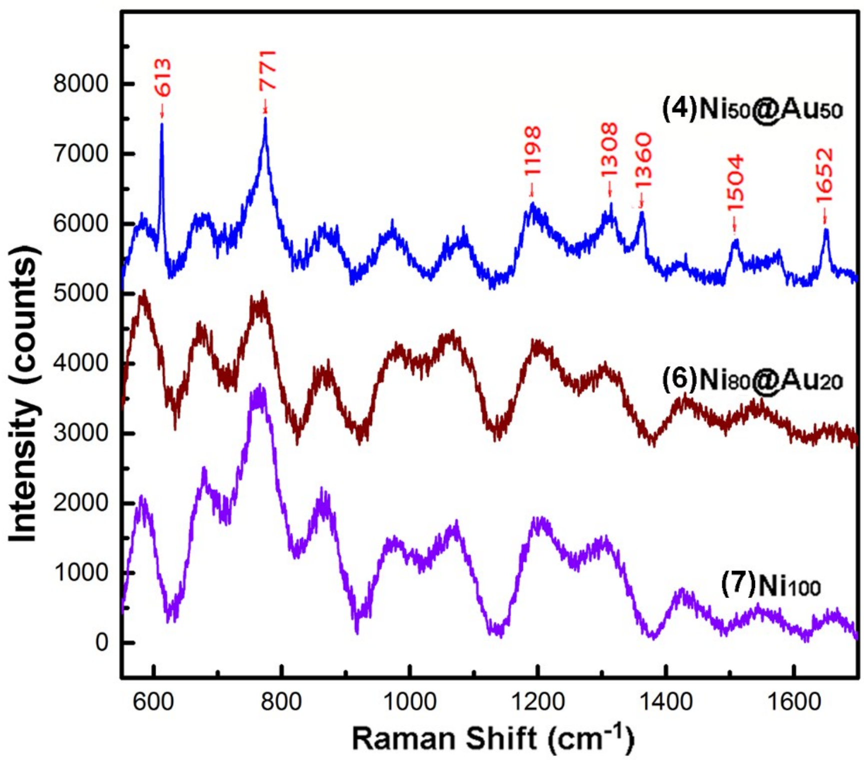

Figure S4. SERS spectra of the 10^{-6} M R6G molecules adsorbed on the substrates of Ni_{100} (7), $Ni_{80}@Au_{20}$ (6) and $Ni_{50}@Au_{50}$ (4), respectively.

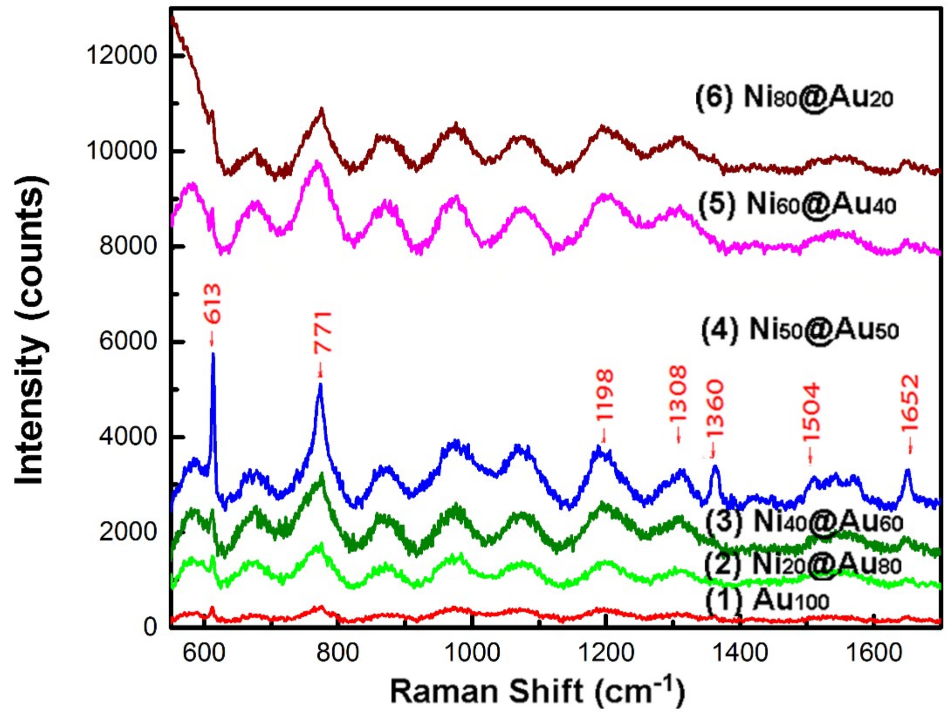

Figure S5. SERS spectra of the 10^{-4} M R6G molecules adsorbed on the substrates of Au_{100} (1), $Ni_{20}@Au_{80}$ (2), $Ni_{40}@Au_{60}$ (3), $Ni_{50}@Au_{50}$ (4), $Ni_{60}@Au_{40}$ (5) and $Ni_{80}@Au_{20}$ (6) NPAs, respectively.
